# Dietary Exposure Estimation to Chemicals Transferred from Milk and Dairy Products Packaging Materials in Spanish Child and Adolescent Population

**DOI:** 10.3390/foods9111554

**Published:** 2020-10-27

**Authors:** Antía Lestido-Cardama, Raquel Sendón, Juana Bustos, Mª Luisa Lomo, Perfecto Paseiro Losada, Ana Rodríguez Bernaldo de Quirós

**Affiliations:** 1 Department of Analytical Chemistry, Nutrition and Food Science, Faculty of Pharmacy, University of Santiago de Compostela, 15782 Santiago de Compostela, Spain; raquel.sendon@usc.es (R.S.); perfecto.paseiro@usc.es (P.P.L.); ana.rodriguez.bernaldo@usc.es (A.R.B.d.Q.); 2 National Food Center, Spanish Agency of Food Safety and Nutrition, E-28220 Majadahonda, Spain; JBustos@mscbs.es (J.B.); MLomo@mscbs.es (M.L.L.)

**Keywords:** exposure estimation, TDS-like investigation, food packaging, dairy products, composite food samples, GC-MS

## Abstract

Packaging materials are subject to risk assessment since they can transfer their components to the food, and they may constitute a risk for the consumers’ health. Therefore, estimating the exposure to chemicals migrating from packaging is required. In this study, a novel approach based on a total diet study (TDS)-like investigation to evaluate the exposure to chemicals transferred from the packaging was presented. The proposed methodology involved a non-targeted gas chromatography coupled to mass spectrometry (GC-MS) method to identify potential migrants and the determination of the migrants in composite food samples. The method was applied to evaluate the dietary exposure to chemicals from food packaging materials used for milk and dairy products in the Spanish child and adolescent populations. Several migrants identified in packaging materials were selected to determine their concentration in composite food samples. These chemicals included diethyl phthalate (DEP), diisobutyl phthalate (DIBP), dibutyl phthalate (DBP), bis(2ethylhexyl) phthalate (DEHP), benzophenone (BP), 1,3-diphenylpropane (1,3-DPP), and bis(2-ethylhexyl) terephthalate (DEHT). The method exhibited a good sensitivity (limit of detection, LOD ≤ 0.05 µg/g) and a satisfactory recovery (78.4-124%). Finally, the exposure was estimated using the Spanish national dietary survey ENALIA. Phthalates DBP and DEHP showed the highest mean exposure, ranging from 2.42 (10–17 years)–4.40 (12–35 months) and 1.35 (10–17 years)–4.07 (12–35 months) µg/kg bw/day for DBP and DEHP, respectively.

## 1. Introduction

Human dietary exposure to chemicals is a priority issue for public health authorities and a key step in risk evaluations. In a Joint Guidance from the Food and Agricultural Organization (FAO), World Health Organization (WHO), and European Food Safety Authority (EFSA), the total diet study (TDS) and total diet study (TDS)-like are promoted as useful approaches and complementary to traditional monitoring programs and are widely used to determine the dietary exposure of a certain population to food contaminants or also to beneficial substances. The main characteristics of a TDS are the following: should be representative of a typical diet, pooling of foods, and foods are analyzed as consumed. However, sometimes, there are deviations from a proper TDS, for instance, those studies that are focused on a certain group of foods, which are mainly responsible for the exposure of a particular substance, or also studies where samples are not analyzed as they are consumed, etc. These approaches should be considered as TDS-like investigations [1].

From the food safety standpoint, the migration of harmful substances from the food contact materials into the food constitutes a hazard for the health of the consumers through dietary exposure.

Among the potential migrants that can be found in packaging materials, phthalates have been a cause for concern due to their adverse effects on human health. They have been reported as endocrine disruptors, and several of them and their metabolites have been described as teratogenic in animals [2,3]. Regarding their applications, even though this group of chemicals is used mainly as plasticizers to provide flexibility to plastics, particularly to polyvinyl chloride (PVC), they can also be present in printing inks and adhesives [2,4]. Some of them, such as dibutyl phthalate (DiBP) and bis(2-ethylhexyl) phthalate (DEHP), among others, are included in the list of additives authorized for plastic food contact materials with restrictions, including specific migration limits (SML) of 0.3 mg/kg and 1.5 mg/kg, respectively [5].

It is interesting to note that although there is a tendency for a decrease of phthalates in food packaging, in the five past years, 21 notifications were reported through the rapid alert system for food and feed (RASFF). In recent years, other plasticizers, including bis(2-ethylhexyl) terephthalate (DEHT), are being used as substitutes to phthalates in food packaging applications. 

Other compounds that have also received particular attention are photoinitiators, which are components of printing inks. In food packaging materials, benzophenone is widely used as an initiator in UV-cured inks. It is included in the European Union positive list of authorized substances for plastic materials and articles intended to come into contact with food as additive with an SML of 0.6 mg/kg [5,6]. 

Very limited information about the exposure to chemicals transferred from food packaging is available in the scientific literature. For example, Sakhi et al. [2] investigated the dietary exposure to phthalates and bisphenol A in the Norwegian population, and Fierens et al. [7] reported data on exposure estimation of phthalates in the Belgian adult population. Results from the UK total diet study on exposure to phthalates have also been published [8]. Data about 1,3-diphenylpropane exposure has been reported as well elsewhere [9]. 

In this paper, a novel methodology based on TDS-like investigation for the dietary exposure estimation of chemicals from food packaging was developed. The method was applied to the packaging of milk and dairy products, and the exposure was estimated in the child and adolescent population. The approach followed included, first, a GC-MS non-target analysis for the identification of migrants in the packaging. Then, a variety of chemicals, previously detected in the food packaging, including phthalates, benzophenone, bis(2-ethylhexyl) terephthalate, and 1,3-diphenylpropane, were selected for quantitation in the food composites and for exposure estimation. 

For dietary exposure purposes, the food group comprising milk and dairy products was selected because they are extensively consumed. For that, composite food samples were prepared, taking into account the consumption data from the national dietary survey ENALIA for the Spanish child and adolescent population [10].

## 2. Materials and Methods 

### 2.1. Reagents and Analytical Standards

Acetonitrile (ACN) and dichloromethane (DCM), both for liquid chromatography, acetic acid (glacial), 100% anhydrous GR for analysis (AAG), n-hexane, and acetone for gas chromatography, electron-capture detector (ECD), and flame ionization detector (FID), methanol for gas chromatography MS, absolute ethanol for analysis, and sodium chloride GR for analysis were from Merck (Darmstadt, Germany).

Chemical standards of butylated hydroxytoluene (BHT) (99%) (CAS 128-37-0), acetyltributyl citrate (ATBC) (99%) (CAS 77-90-7), triacetin (≥99%) (CAS 102-76-1), bis(2-ethylhexyl)phthalate (DEHP) (99%) (CAS 117-81-7), and bis(2-ethylhexyl) adipate (DEHA) (≥99%) (CAS 103-23-1) were obtained from Fluka (Steinheim, Germany). Diethyl phthalate (DEP) (99.5%) (CAS 84-66-2), diisobutyl phthalate (DIBP) (99%) (CAS 84-69-5), dibutyl phthalate (DBP) (99%) (CAS 84-74-2), bis(2-ethylhexyl) terephthalate (DEHT) (≥96%) (CAS 6422-86-2), benzophenone (BP) (99%) (CAS 119-61-9), toluene-2,6-diisocyanate (97%) (CAS 91-08-7), 2,6-di-tert-butyl-1,4-benzoquinone (98%) (CAS 719-22-2), methyl palmitate (97%) (CAS 112-39-0), glyceryl trioctanoate (≥99%) (CAS 538-23-8), 13-docosenamide (>85%) (CAS 112-84-5), caprolactam (99 + %) (CAS 105-60-2), 2,4-di-tert-butylphenol (99%) (CAS 96-76-4), octocrylene (97%) (CAS 6197-30-4), and squalene (≥98%) (CAS 111-02-4) were supplied by Sigma-Aldrich (Schnelldorf, Germany). Hexadecanamide (95%) (CAS 629-54-9) and 1,3-diphenylpropane (1,3-DPP) (98%) (CAS 1081-75-0) were purchased from Combi-Blocks (San Diego, CA, USA). Toluene-2,4-diisocyanate (CAS 584-84-9) and 4,4′-diphenylmethane diisocyanate (CAS 101-68-8) were obtained from Merck (Darmstadt, Germany). Internal standard diethyl phthalate-3,4,5,6-d4 (CAS 93952-12-6) was obtained from Fluka (Steinheim, Germany). 

For calibration purposes, individual standard solutions of 1,3-DPP, DIBP, DBP, DEHP, and DEP were prepared in methanol, and for BP and DEHT, the solutions were prepared in ethanol and acetone, respectively. All solutions were at a concentration of 1000 mg/L. Diluted solutions were prepared with ACN:DCM (50:50 *v*/*v*) within the range of 0.005–2.5 mg/L, and all of them contained the internal standard prepared in ACN at a final concentration of 0.5 mg/L. Standard solutions were stored at 4 °C in the dark.

### 2.2. Samples

A total of fourteen food items within the milk and dairy products category were included in the study. Foods were selected to be representative of the Spanish child and adolescent population diet according to the Spanish dietary survey ENALIA [10]. Food samples of different brands were purchased in a local supermarket. Food samples were subjected to freeze-drying in a lyophilizer (Telstar LyoQuest, Tokyo, Japan) till dry powder was obtained (−70 °C, 0.006 mBar, 72 h) and stored in the freezer until analysis. 

The packaging materials were analyzed in order to identify potential migrants. First, an identification of the polymers by FTIR was made. The thickness of the packaging was measured with a manual digital micrometer (Mitutoyo-Japan, Kanagawa, Japan). The data presented were the average of three measurements. Detailed information about both food samples and packaging materials is summarized in Table 1.

### 2.3. Equipments and Analytical Conditions

**Figure 1:**
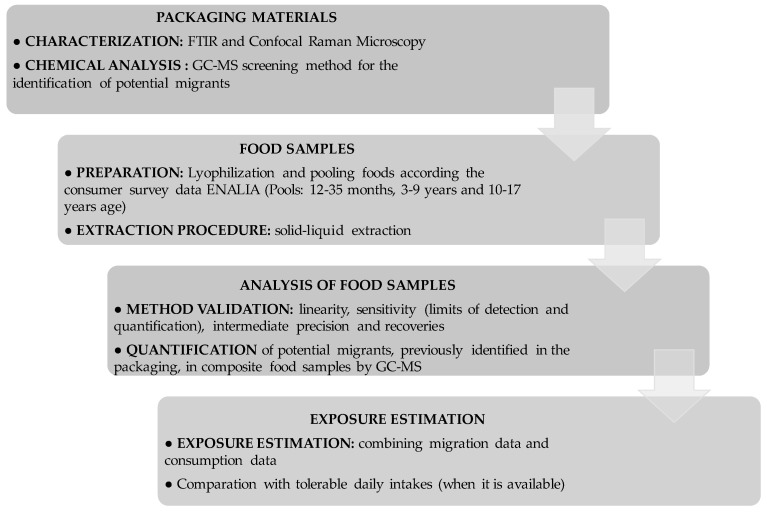
Scheme of the analytical protocol followed in this study.

A scheme of the analytical protocol followed is presented in Figure 1.

#### 2.3.1. Fourier-Transform Infrared Spectroscopy (FTIR)

To identify the type of material, infrared spectra were acquired using an ATR (attenuated total reflectance)-FTIR spectrometer (FT-IR 4700, Jasco, Japan) equipped with a diamond optical crystal and controlled by Spectra Manager^TM^ Suite software (Jasco, Japan). FTIR spectra were acquired in the range 4000–650 cm^−1^, performing a total of 25 scans with a resolution of 4.0 cm^−1^.

#### 2.3.2. Confocal Raman Microscopy

Measurements were performed using a WITec confocal Raman microscopy alpha300 R (WITec GmbH, Ulm, Germany) coupled to an Ultra-High-Throughput-Spectrometer UHTS300 for visible, equipped with a back-illuminated CCD camera with a quantum efficiency > 90% (500–700 nm). The equipment characteristics and data processing software are described elsewhere [11]. Each packaging was investigated by performing an x-z scan with a scan range of 90 × 200 µm^2^, 150 × 150 pixels (22,500 spectra), and 1 ms/spectrum acquisition time. The excitation source was a diode laser with an emitting wavelength of 532 nm. Laser power was adjusted to 25 mW. The spectra identification was performed by using WITec True Match Database Management software to compare the sample spectra obtained with the commercial database ST Japan, which includes a total of 3412 Raman spectra of polymers and polymer additives.

#### 2.3.3. Gas Chromatography Coupled to Mass Spectrometry (GC-MS)

GC-MS analysis was carried out according to the method described by García Ibarra et al. [12] on a Thermo Scientific Trace 1300 Series Gas Chromatograph (Thermo Fisher Scientific, San José, CA, USA) equipped with a Trace ISQ LT mass detector and an AI 1310 autosampler. For screening purposes, a ZB-5MS (30 m × 0.25 mm × 0.25 µm) column was used as a stationary phase. For the quantification of the selected analytes (1,3-DPP, DIBP, DBP, DEHP, DEP, BP, and DEHT), injections were performed in split mode (1:5), the oven ramp temperature started at 60 °C, and data were acquired in SIM mode using the target and qualifier ions presented in Table 2; otherwise, the conditions were as specified elsewhere [12].

#### 2.3.4. Preparation of Food Samples and Exposure Estimation

Composite food samples were prepared by pooling food items to be representative of the Spanish child and adolescent population diet. To estimate the dietary exposure, the GEMS/Food–EURO recommendations were followed; thereby, for analytical results lower than the limit of quantification (<LOQ, non-quantifiable), values equal to LOQ/2 were considered [13].

#### 2.3.5. Extraction Procedure-Foodstuffs

Seven chemicals previously identified in packaging materials, namely, DEP, BP, 1,3-DPP, DIBP, DBP, DEHP, and DEHT, were selected to investigate their migration into the food, and therefore their concentration in the food samples was determined. A composite food sample prepared, as described above, was used to optimize the extraction procedure. The proposed method is based on that reported by Bradley et al. [8] with some modifications.

Briefly, one gram of the lyophilized composite food sample was weighed in a glass centrifuge tube (a known concentration of standards was added and allowed to stand for 15 min before extraction). Then, 10 mL of ACN:DCM (50:50 *v*/*v*) and 1 mL of glacial acetic acid were added, and the samples were shaken manually for 15 min, followed by centrifugation (3500 rpm, 10 min, −5 °C). The supernatant was removed and stored at (−30 °C) for 24 h. After the addition of NaCl, the extracts were vortexed (VELP scientifica vortex). An aliquot of 10 mL was evaporated until dryness under a stream of nitrogen (RapidVap Vertex Evaporator, Labconco). One milliliter of ACN with 500 µL of the internal standard (DEP-d 0.5 µg/mL) was used to dissolve the residue. The resulting extract was filtered with a 0.45 µm PTFE filter.

In developing the method, different solvents, including acetonitrile, acetonitrile:dichloromethane (1:1 *v*/*v*), hexane, and acetonitrile:dichloromethane (1:1 *v*/*v*) with acetic acid, were assayed. Hexane yielded low recoveries (47.9–52.2%) for DIBP, DBP, DEHP, and DEHT; poor recoveries (<50%) were also obtained for DEHP and DEHT when using acetonitrile:dichloromethane (1:1 *v*/*v*). Moreover, acetonitrile gave neither good results for DEHT (recovery < 57%). Thus, acetonitrile:dichloromethane (1:1 *v*/*v*) with acetic acid proved to be the most appropriate extraction solvent for all analytes.

NaCl was added to allow breaking the emulsion. After the extraction and addition of NaCl, the extract was evaporated to dryness or to a given volume (<1 mL). In this last case, the results were not quantitatively reproducible; therefore, evaporation to dryness was performed in subsequent analysis.

Most of the methods described in the literature involve clean-up steps with solid-phase extraction (SPE), QueChers, and so on; in order to minimize the risk of contamination with phthalates, these steps were not considered in this work.

#### 2.3.6. Quality Assurance/Quality Control (QA/QC)

Validation parameters, such as linearity, sensitivity (limits of detection and quantification), intermediate precision, and recoveries, were addressed.

Quantification was performed on the basis of linear calibration plots of peak area/internal standard area ratio against concentration. Calibration curves were constructed using at least five concentration levels in the range from LOQ to 2 or 2.5 μg/g. The limits of detection and quantification, defined as a signal three and ten times, respectively, and the height of the noise level were determined according to the Analytical Chemical Subcommittee guidelines [14].

Intermediate precision and recoveries were determined by spiking a composite food sample with known amounts of the analytes at three different concentration levels (0.25; 0.5; 1 µg/g) and in three separate days (six replicates). For that purpose, the pooled sample corresponding to the age group of 3–9 years was used.

To minimize the contamination with phthalates, the plastic material was avoided, and the glassware was heated at 400 °C for two hours in a muffle oven (Nabertherm GmbH, Lilienthal/Bremen, Germany).

## 3. Results and Discussion

### 3.1. Characterization of Packaging Materials by FTIR and Confocal Raman Microscopy

The internal and external side of the packaging materials were identified by FTIR using KnowItAll^®^ 17.4.135.B IR Spectral Libraries of Polymers and Related Compounds (Bio-Rad Laboratories, Inc., Hercules, CA, USA). Results are shown in Table 1. Polyethylene was the most common polymer identified in the analyzed samples.

**Figure 2:**
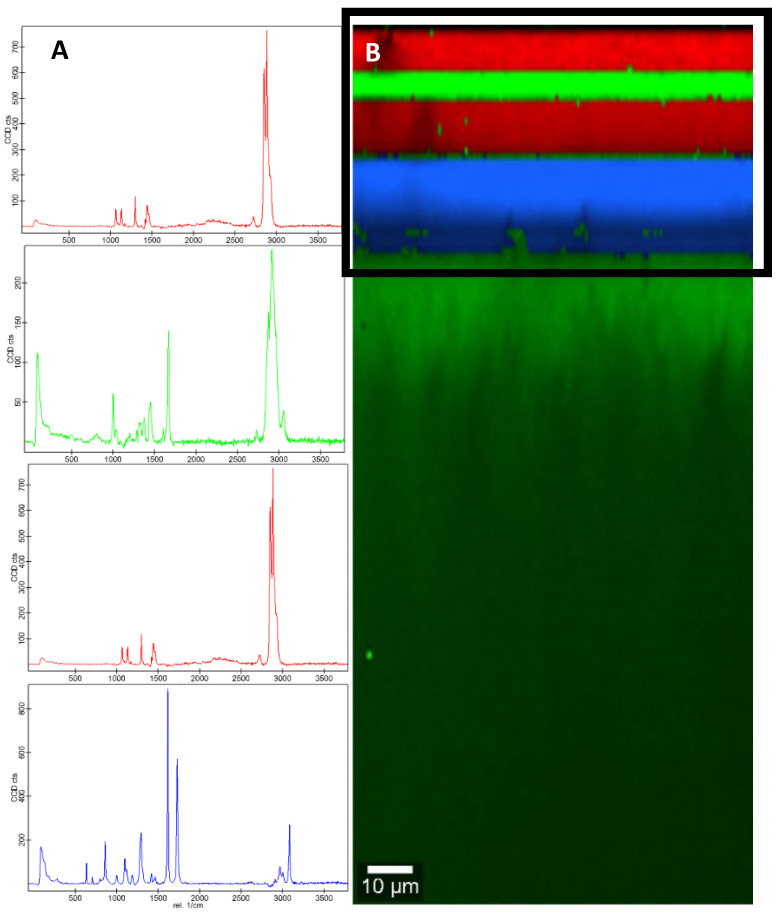
Raman spectra (**A**) and corresponding color-coded Raman image (**B**) of the lid of the semi-cured cheese sample.

Confocal Raman microscopy provided a three-dimensional characterization of the sample by imaging the different layers, as can be seen in Figure 2. In addition, within the acquired multi-spectrum file, each spectrum was identified with the library. A sample of each food group was analyzed by this technique.

For the cheese group, sample QS01 was selected, and both the packaging and the lid were analyzed. In the lid, four different spectra were observed (Figure 1): the red spectra, which corresponds to the internal side, was identified as low-density polyethylene (LDPE), then the green spectra turned out to be styrene/isoprene copolymer, another layer of polyethylene is repeated, and the blue spectra, which corresponds to the external side, was identified as PET film. In the case of the packaging, only two layers were visualized, an internal one that was identified as LDPE and an external one as PET. The reverse signals were obtained by scanning these samples in the opposite direction to confirm the results obtained. The identification of both the external and internal layers coincided with the identifications made in the IR-ATR (Table 1) for these same samples for both the packaging and the lid.

Within the milk group, sample LS01 was selected, but due to its thickness (430 µm), the laser was not able to cross it, and only the outermost layer could be identified. The internal side was identified as LDPE and an external one a PET base, and different pigments were used for inks, which coincided with the results obtained in the IR-ATR. Finally, the packaging of the yogurt YN01 and the flan FN01 belonging to the group of dairy desserts was selected. The lids were discarded in this case because they contained aluminum, which reflected the Raman signal. In the flan packaging, because of the thickness (667 µm), only the outermost layer could be identified, being both isotactic PP as in the results of IR-ATR. In the yogurt packaging, both sides were made of polystyrene of high impact as in IR-ATR with titanium dioxide used in white inks.

### 3.2. Identification of Potential Migrants in Packaging Materials

With the aim of identifying potential migrants from the food packaging materials, a non-targeted analysis was performed using GC-MS. Acetonitrile extracts from packaging samples were analyzed.

More than 90 compounds were detected, and NIST/EPA/NIH 11 Mass spectral library (version 2.0) and Wiley Registry^TM^ 8th (UK) were used for the identification. Moreover, 21 compounds were confirmed by comparison with standards, and the remaining peaks were considered to be tentatively identified.

An in silico model, specifically Toxtree v2.6.13 (Ideaconsult Ltd., Sofia, Bulgaria) software, was used to predict the toxicity of the identified substances. This tool classified the molecules into three classes of toxicity (low, intermediate, high) according to their chemical structure.

Both confirmed and tentatively identified compounds are listed in Table 2. Considering that, in general, a direct matching factor (SI) and a reverse search matching factor (RSI) of 900 or greater is an excellent match, and 800–900 is a good match, solely compounds with matching factors SI and RSI higher than 800 were included.

Many substances of different chemical nature, including aldehydes, ketones, carboxylic acids, alcohols, and so on, were tentatively identified in the analyzed samples. 2-Heptanone was found only in one sample (LE01), and this compound has been identified in paper and board samples [15]. Benzaldehyde and benzoic acid are included in the European Union positive list of substances authorized in plastic materials [5]. They were found in eight (LE01, LE02, LS01, LS02, QF02_P, YN01_P/L, YS01_P/L, FN03_P/L) and three samples (YN01_P/L, YS01_P/L, FN02_P), respectively. Other compounds identified were 4-methyl-2-heptanone, phenylacetaldehyde, 2-nonanone, and 1-dodecanol.

Four diisocyanates, namely 2,6-toluene diisocyanate, 2,4-toluene diisocyanate, isophorone diisocyanate, and 4,4′-diphenylmethane diisocyanate, were detected in several samples, including cheese (QS01_L, QL01_E, QF_02L), yogurt (YN01_L, YS01_L), and dairy desserts (FN02_P, FN03_L) packaging. They are widely used to produce polyurethane adhesives. All of them are classified as III class according to Cramer rules. 1-(2-Methoxypropoxy)-2-propanol was found in two samples (LE02, LS02), and this compound has also been identified in polyurethane adhesives [16].

Abietic acid and their derivatives, such as retene, dehydroabietal, methyl dehydroabietate, and dehydroabietic acid, were found in different samples. These compounds have been reported in hotmelt adhesives [17]. Retene belongs to class III according to Cramer rules and was detected only in one sample (LE01); dehydroabietal was identified in five samples—LE01, LE02, LE03, LS01, and LS02, and methyl dehydroabietate was found in samples LE01, LE02, LE03, LS01, LS02, YN01_L, YS01_L, FN02_P, and both belong to class II. Dehydroabietic acid and abietic acid were detected in three samples, YN01_L, YS01_L, and FN03_L, and presented intermediate and high toxicity, respectively.

Styrene, a monomer widely used in the plastic industry, was identified in eight samples (LS01, LS02, QS01_I, QF02_P, YN01_P, YS01_P, FN01_L, FN03_P/L). 1,2-Diphenylpropane and 1,3-diphenylpropane were identified in samples YN01_P, YS01_P, QS01_I, QF02_P, YN01_P, YS01_P, FN03_P, respectively. These two compounds with high toxicity (Cramer class III) have been reported in polystyrene-based materials—1,2-diphenylpropane, a thermal degradation product of polystyrene, and 1,3-diphenylpropane, an isomer of the styrene dimers [18,19]. A metabolite of styrene, specifically styrene-7,8-oxide, was detected in only one sample, QF02_P, and the in vitro studies have shown that this compound is carcinogenic [20].

Caprolactam belongs to Cramer class III, and it was identified in eleven samples, comprising milk, cheese, yogurt, and dairy dessert packaging (LE01, LE02, LS01, LS02, QF01, QF02_L, YN01_P/L, YS01_L/P, FN01_P/L, FN02_P, FN03_P/L). It is employed as a monomer in the manufacture of polyamide, and it has been also identified as a residue from printing inks [21]. A caprolactam cyclic dimer, 1,8-diazacyclotetradecane-2,9-dione, was identified in the sample QF01.

Bis(2-hydroxyethyl) terephthalate, a monomer intermediate in the synthesis of PET, was identified in one sample QS01_P [22].

Phthalic anhydride was found in two samples of dairy dessert packaging (FN01_L, FN03_P); this substance is authorized as a monomer and additive in the manufacture of plastic materials. 1,1′-Oxydi-2-propanol was detected in milk packaging (LE01, LE02, LE03, LS01, and LS02); its use is also authorized as an additive and monomer in plastic materials. Both compounds have high toxicity (class III).

Butylated hydroxytoluene (BHT), a synthetic phenolic antioxidant, authorized as an additive in plastic food contact materials with an SML of 3 mg/kg was identified in 13 samples (LE01, LE02, LE03, LS01, LS02, QS01_L/P/I, QL01_I, QF02_L/P, YN01_L, YS01_L, FN01_L, FN02_P/L, FN03_P/L). In addition to its use as a polymeric additive, it is employed as a food additive. In three of the eleven samples, specifically QS01_L, QL01_I, and FN02_L, a metabolite of BHT, namely 2,6-di-tert-butyl-4-methylene-2,5-cyclohexadienone, was detected. 3,5-Di-tert-butyl-4-hydroxybenzaldehyde, another derived metabolite of BHT, was only detected in one sample (FN02_L). The antioxidants, as well as their degradation products or metabolites, have attracted particular attention owing to their potential negative effects as a result of their toxicity [23].

Several compounds that could be considered non-intentionally added substances (NIAS) have been identified in the analyzed samples. For example, 2,4-di-tert-butylphenol, a degradation product of the antioxidants Irgafos^®^ 168 and Irganox^®^ 1010, was detected in eleven samples (LE01, LE03, LS01, LS02, QL01_E, QF01, YN01_P/L, YS01_P/L, FN01_P, FN02_L, FN03_L), and 2,6di-tert-butyl-p-benzoquinone, which has also been reported as a degradation product of the antioxidants Irganox 1010, Irgafos 168, and Irganox PS 802, was found in eight samples (LE03, LS02, QS01_L, QF02_L/P, YN01_P/L, YS01_P/L, FN01_P/L, FN03_P/L) [24]. Both compounds present different toxicity; while the first is classified as class I (low toxicity), the second one is classified as class II (intermediate toxicity). Another degradation product of the antioxidant Irganox^®^ 1010, 7,9-di-tert-butyl-1-oxaspiro[4.5]deca-6,9-diene-2,8-dione, was found in all samples (LE01, LE02, LE03, LS01, LS02, QS01_L, QL01_E, QF01, QF02_L/P, YN01_P/L, YS01_P/L, FN01_P, FN02_L, FN03_P/L), and this compound is classified in class III according to Cramer rules.

3,6,9,12,15-Oxabicyclo(15,3)heneicosa-1(21),17,19-triene-2,16-dione was only found in one sample (FN01_L); this substance has been reported as an antioxidant degradant and belongs to class III according to Cramer rules. Diphenylmethane, which has been described as a monomer degradant, was detected in three samples (YN01_L, YS01_L, FN03_ L) and similarly presents high toxicity [24].

In two samples of packaging cheese (QS01_I, QF02_P) and in one sample corresponding to the packaging of dairy desserts (FN03_P), trans-1,2-diphenylcyclobutane was identified. In a study conducted by Lago and Ackerman [24], this substance has been reported as a monomer byproduct.

Diethylene glycol monoethyl ether was found in milk packaging samples (LE01, LE02, LE03, LS01, LS02); this compound has been described by Bentayeb et al. [25] in a study in which the authors investigated the set-off phenomenon of photoinitiators in food packaging materials by using direct analysis in real-time coupled to time-of-flight mass spectrometry (DART/TOF-MS). It was tentatively identified as a set-off compound other than photoinitiators.

One of the other print-related compounds identified in some of the samples analyzed (QF01_P, YN01_P, YS01_P, and FN03_P) was 1,1-diphenylethylene, which has been classified as high toxicity substance [24].

Benzophenone was detected in six samples—LE01, LE02, LE03, LS01, LS02, and QF02_L/P; this compound has also high toxicity and belongs to class III according to Cramer rules.

Several phthalates, including diethyl phthalate (DEP) (LE01, LE02, LE03, LS01, LS02, QS01_-/P/I, QF02_L/P, YN01_P/L, YS01_P/L, FN01_P/L, FN02_L, FN03_P/L), diisobutyl phthalate (DIBP) (LE01, LE02, LE03, LS01, LS02, QF02_P, YN01_P/L, YS01_P/L, FN01_P/L, FN02_P/L, FN03_P/L), dibutyl phthalate (DBP) (LE01, LE02, LE03, LS01, LS02, YN01_L, YS01_L), and bis(2-ethylhexyl) phthalate (DEHP) (LE03, LS01, QS01_P, QL01_E, QF01, QF02_L YN01_L, YS01_L, FN01_P/L, FN02_P/L, FN03_L), among others, were found in different samples. These compounds, besides plasticizers, can be found in printing inks formulations and also have been employed as solvents to hold color [3,26].

Others common plasticizers, such as acetyltributyl citrate (ATBC) (QS01_L, QL01_E/I, QF01, QF02_L, YN01_P/L, YS01_P/L, FN01_L, FN02_P/L, FN03_L) and diethylhexyl adipate (DEHA) (LE01, LS01, LS02, LE03, YN01_L, YS01_L, FN03_P/L), were detected in different material samples; these substances are authorized as an additive in plastic food contact materials. 2-Ethyl-1-hexanol, the alcoholic component of DEHA, was identified in the sample FN03_P [27].

Squalene was detected in eleven substances (LE01, LE02, LS01, LS02, QS01_P/I, QL01_I, QF01, QF02_L/P, YN01_P/L, YS01_P/L, FN01_P/L, FN02_P/L, FN03_P/L). This compound chemically is a hydrocarbon. One of its uses is as a plasticizer. On the other hand, triacetin was identified in three samples (QL01_E, YN01_L, YS01_L), besides its use as a food additive; this compound has been described as an eco-friendly plasticizer [28].

Lubricants, such as isopropyl laurate and glyceryl tricaprylate, were found in samples FN02_L and QS01_P, respectively.

Two slip agents—erucamide and hexadecanamide—were identified in six (QS01_L/P, QL01_E/I, QF01, FN01_P, FN02_P/L, FN03_P/L) and seven samples (QS01_P, QL01_E, YN01_L, YS01_L, FN01_P, FN02_L, FN03_L), respectively; both compounds are classified in class III according to Cramer rules.

Other compounds classified in class III (high toxicity), including 2,5-dimethyl-2,5-hexanediol, 1,1,3-trimethyl-3-phenylindan, 2,3-dimethyl-2,3-diphenylbutane, 1-phenylnaphthalene, and (1-methyl-2,2-diphenylcyclopropyl) sulfanylbenzene, were identified in several samples.

### 3.3. Analytical Parameters

The optimized method was validated regarding linearity, sensitivity (limits of detection and quantification), intermediate precision, and recoveries.

Each point of the calibration curve was injected by triplicate (Table 3). All compounds exhibited appropriate linearity with *r* ≥ 0.9900.

The LODs obtained in this study (Table 3) for phthalates were comparable to those reported by Bradley et al. [4]. Regarding the LOD of DEHT, our result was slightly lower than that obtained by Lo Turco et al. [29].

Precision values obtained, expressed as the % RSD, were <18.5%. Bradley et al. (2013) [4] reported repeatability values (RSD%) ranging from 3.6–15.8% in a cheese sample spiked at a concentration of 50 μg/kg. The recoveries ranged from 78.4% to 124% (Table 3). These recovery values were comparable to those reported by Bradley et al. [4] for phthalate diesters in foods (71.6–116.3%). On the other hand, the values were similar to those described by Cao et al. (2015) [30] in composite food samples and slightly higher than those reported by Jia et al. (2014) [31] in milk samples (94.9–99.4%).

### 3.4. Migrants Concentration in Food and Dietary Exposure Estimation

Foods were grouped in three pools for each subgroup of the population, namely 12–35 months, 3–9 years, and 10–17 years, and they were prepared, as described above. The concentrations of the analytes studied in the food composite samples are presented in Table 4. Samples were analyzed in duplicate (average ± SD). All of the phthalates studied, that is, DEP, DIBP, DBP, and DEHP, were found in the three pools. In general, the pool that corresponds to the adolescent group was the one that presented the highest values ranging from 0.0317 to 0.1627 µg/g. Among the phthalates, DBP, followed by DEHP, were found at higher concentrations. Van Holderbeke et al. [32] reported DEHP as the most frequent phthalate in the milk and dairy products group, and moreover, in another work conducted by the same authors [7], it was found that this food group was the one that contributed the most to the exposure to DBP.

However, the other analytes studied—BP, 1,3-DPP, and DEHT—were found below LOQ in all composite food analyzed samples.

The concentrations of phthalates reported in this work were generally higher than those described by Sakhi et al. [2] in Norwegian foods and beverages, except in the case of DEHP, in which they found higher values in different samples of cheese. Van Holderbeke et al. [32] also found higher levels of DEHP in milk and dairy products sold in the Belgian market.

Nevertheless, Jia et al. [31] reported lower concentrations for DEP (13 µg/kg) and DEHP (57 and 42 µg/kg) in milk and yogurt samples.

Values reported in a TDS carried out in Canada (2013) were lower than those found in our study for DEP, DIBP, and DBP, whereas in the case of DEHP, they found higher concentrations in some of the dairy products [30].

Cariou et al. [33] used GC-MS to determine four phthalates (DIBP, DBP, BBzP, and DEHP) in different food items. The concentrations determined in whole milk were <2.7 ng/g for DIBP, 0.5 ng/g for DBP, and 21.8 ng/g for DEHP, and in concentrated milk samples, the values were 2.9, 0.4, and 25.5 ng/g for DIBP, DBP, and DEHP, respectively. These values were also lower than those reported in the present study.

Exposure to contaminants through the diet is one of the essential elements in risk evaluations. The exposure to the selected chemicals previously identified in the packaging materials was investigated. The dietary exposure (mean and 95th percentile) for the different age groups is summarized in Table 4.

Migration from food packaging seems to be one important source of exposure to phthalates. Estimated mean exposure to DBP and DEHP was quite similar; values ranged from 2.42 µg/kg bw per day (pool 10–17 years) to 4.40 µg/kg bw per day (pool 12–35 months) for DBP and from 1.35 µg/kg bw per day (pool 10–17 years) to 4.07 µg/kg bw per day (pool 12–35 months) for DEHP. These values were higher than those calculated for DIBP and DEP. The lowest exposure was found for DEP, and the values varied between 0.472 µg/kg bw per day (pool 10-17 years) and 1.19 µg/kg bw per day (pool 12–35 months). The estimated 95th percentile exposure varied from 6.54 µg/kg bw per day (pool 10–17 years) to 21.3 µg/kg bw per day (pool 12–35 months) for DEHP and from 2.29 µg/kg bw per day (pool 10–17 years) to 6.24 µg/kg bw per day (pool 12–35 months) for DEP. It is interesting to note that, in general, the dietary exposure decreases with increasing age. This can be explained in part due to an increase in body weight with age. Sirot et al. [34] observed a similar trend in a study on the exposure to acrylamide in the diet of the French population.

For certain phthalates, the EFSA has established tolerable daily intakes (TDI); for those of interest in this study, the TDIs were 0.01 mg/kg bw per day for DBP and 0.05 mg/kg bw per day for DEHP. In the case of DEP, the World Health Organization (WHO) [35] specifies a TDI of 0.5 mg/kg bw per day (WHO, 2003). Recently, the EFSA Panel on Food Contact Materials, Enzymes, and Processing Aids (CEP Panel) [36] at the request of the European Commission has updated the establishment of the risk of DBP, BBP, DEHP, DINP, and DIDP. In a draft update published, the CEP panel has re-confirmed the individual TDI derived in 2005 for all the phthalates but also proposes a group-TDI for DEHP, DBP, and BBP and establishes a value of 50 μg/kg bw per day, expressed as DEHP equivalents.

In examining our data (Table 4) and considering the individual TDI, the estimated exposure for all age groups was below the TDI, except for DBP at the 95th percentile, which exceeded the TDI up to 2.3 times in the 12–35 months age group. In line with this result, Cirillo et al. [37] studied neonatal exposure to phthalates and bisphenol A and also found that the daily intake of DBP exceeded TDI up to 175%.

In a study carried out by Sakhi et al. [2], the estimated dietary exposure to phthalates in the Norwegian adult population was examined. The results revealed that DEHP and DiNP presented the highest mean estimated dietary exposure with values ranging from 396 to 436 ng/kg bw/day and from 477 to 494 ng/kg bw/day for DEHP and DiNP, respectively.

Fierens et al. [7] used a semi-probabilistic modeling approach for dietary phthalate exposure in the Belgian adult population. The phthalates considered in this study were DEP, DBP, BBP, and DEHP. Of all the phthalates investigated, DEHP showed the highest predicted exposure value (1.45 µg/kg bw/day). These results were lower than those reported by Sakhi et al. [2] for the Norwegian population.

Dietary exposure to DEHP in the Chinese population was investigated by Sui et al. [38]. The mean dietary exposure values found were 4.51, 3.41, 2.46, and 2.03 µg/kg bw per day for the 2–6 years, 7–12 years, 13–17 years, and ≥ 18 years, respectively. The average value for the 2–6 years group was twice higher than that determined in this work for the 3–9 years group. This difference could be attributed partly to an increase in body weight with age and also due to the food products included in the study of Sui et al. [38].

Results from the UK TDS (total diet study) have shown that DEHP is the phthalate that provides the highest exposure, being dairy products, fish, and milk the main contributors for toddler (1.5–2.5 years) subgroup and meat and dairy products for toddler (3.5–4.5 years) subgroup. At 97.5th percentile, the values range from 5.7 to 9.9 µg/kg bw/day for toddlers, between 2.7 and 6.7 µg/kg bw/day for young people, and from 3.4 to 4 µg/kg bw/day for adults [8].

Phthalate intake in the Belgian preschool children population was investigated by Sioen et al. [39]. The dietary exposure was calculated by using the Monte Carlo risk assessment program (MCRA); the authors found that the highest intakes corresponded to DEHP, followed by DiBP and DBP.

In the present study, if we take into account the exposure to DEHP, DBP, and DiBP, a total mean dietary exposure due to the sum of these three phthalates was estimated to be 4.0 µg/kg bw/day (pool 10–17 years), 4.6 µg/kg bw/day (pool 3–9 years), and 6.9 µg/kg bw/day (pool 12–35 months). In all pools, a group-TDI lower than 50 µg/kg bw/day was determined [36].

Other substances identified in packaging materials, specifically BP, 1,3-DPP, and DEHT, were also considered in this study. Regarding BP, the average dietary exposure ranged from 0.744 µg/kg bw per day (pool 10–17 years) to 2.25 µg/kg bw per day (pool 12–35 months), in all cases, was lower than the TDI (0.03 mg/kg bw per day) specified by EFSA [6]. The estimated dietary exposure to DEHT was also below TDI (1 mg/kg bw) in all age groups [40]. For 1,3-DPP, the estimated dietary exposure ranged from 0.372 µg/kg bw per day (pool 10–17 years) to 1.13 µg/kg bw per day (pool 12–35 months).

Although, in general, low exposure data are obtained; however, it is important to consider that consumers are exposed to different chemicals through the diet from several sources; accordingly, there is combined exposure to multiple chemicals (cumulative exposure) and also exposure to a certain substance from several sources (aggregate exposure) [41]. Therefore, it is necessary to take into account all these aspects to evaluate the possible effects arising from exposure to multiple chemicals in foods.

## 4. Conclusions

In brief, a novel analytical approach based on TDS-like investigations was developed, with the aim of estimating the dietary exposure to chemicals from food packaging materials.

The proposed approach comprised a screening step to identify potential migrants in the packaging and the determination of the chemicals in the food composite samples to later estimate the dietary exposure. In this study, the methodology was applied to the packaging of milk and dairy products. The dietary exposure to different packaging contaminants, including phthalates, BP, 1,3-DPP, and DEHT, was investigated in the child and adolescent population. As far as we know, very limited data on exposure to packaging contaminants, particularly to 1,3-DPP and DEHT, has been reported. Although, in general, our data suggested a low dietary exposure to the contaminants evaluated, with the exception of DBP at the 95th percentile that exceeded the TDI; it is important to consider the combined exposure to multiple chemicals and possible synergistic effects for risk assessment determinations.

## Figures and Tables

**Table 1:** Summary of the food samples included in the study, fat content, type of packaging material, and thickness.

Code	Type of Sample	Part of the Sample	Type of Material		Thickness (µm)	Fat Content
			Internal Side	External Side		
LE01	Whole milk	Packaging (P)	PE	PE	433 ± 0.6	3.6 g/100 mL (Satur. 2.5 g)
LE02	Whole milk	Packaging	PE	PE	431 ± 0.6	3.6 g/100 mL (Satur. 2 g)
LE03	Whole milk	Packaging	PE	PE	296 ± 1.0	3.6 g/100 mL (Satur. 2.5 g)
LS01	Semi-skimmed milk	Packaging	PE	PE	430 ± 0.6	1.6 g/100 mL (Satur. 1.1 g)
LS02	Semi-skimmed milk	Packaging	PE	PE	454 ± 1.2	1.9 g/100 mL (Satur. 1.1 g)
YN01	Natural yogurt	Lid (L)	PES	NC	59 ± 1.0	2.9 g/100 mL (Satur. 1.8 g)
		Packaging	PS	PS	252 ± 2.0	
YS01	Strawberry yogurt	Lid	PES	NC	65 ± 0.6	1.9 g/100 g (Satur. 1.1 g)
		Packaging	PS	PS	245 ± 2.0	
FN01	Egg flan with caramel	Lid	SBI	NC	106 ± 1.2	1.8 g/100 g (Satur. 0.6 g)
		Packaging	PP	PP	667 ± 1.5	
FN02	Egg flan	Lid	MP	PES	73 ± 0.6	3.2 g/100 g (Satur. 1.2 g)
		Packaging	VP	PR	186 ± 1.2	
FN03	Custard	Lid	PES	NC	70 ± 0.6	3 g/100 g (Satur. 1.8 g)
		Packaging	PS	PS	300 ± 1.0	
QS01	Semi-cured cheese	Lid	PE	PES	90 ± 1.0	35 g/100 g (Satur. 24 g)
		Packaging	PE	PET	272 ± 1.2	
		Intermediate sheet	PS	PS	56 ± 0.6	
QL01	Molten cheese	External packaging (E)	PE	PES	51 ± 0.6	13.5 g/100 g (Satur. 9 g)
		Internal packaging (I)	PE	PP	25 ± 0	
QF01	Mozzarella	Packaging	PE	Nylon (PA)	70 ± 0.6	18 g/100 g (Satur. 13 g)
QF02	Pasteurized cheese	Lid	PES	PES	38 ± 1.0	14 g/100 g (Satur. 9.6 g)
		Packaging	PS	SB	162 ± 1.5	

MP: Copolymer methylmethacrylate-stat-butylmethacrylate; NC: Nitrocellulose; PA: Polyamide; PE: Polyethylene; PES: Polyester; PET: Polyethylene terephthalate; PP: Polypropilene; PR: Phenoxy resin; PS: Polystyrene; SB: Styrene-butadiene copolymer; SBI: Styrene-butadiene-isoprene rubber; VP: Copolymer vinyl chloride/vinyl acetate.

**Table 2:** Compounds identified in packaging materials and their level of toxicity (TC) according to Cramer rules.

TR (min)	CAS No.	Compound Name	TC	Ion	Milk	Cheese	Yogurt and Dairy Desserts
6.25	110-43-0	2-Heptanone	II	43, 58, 71	LE01		
6.29	100-42-5	Styrene	I	104, 78, 51	LS01, LS02	QS01_I, QF02_P	YN01_P, YS01_P, FN01_L, FN03_P/L
6.62	497-23-4	2(5H)-Furanone	III	55, 84, 39			YN01_L, YS01_L
6.80	98-82-8	Cumene	I	105, 120, 79		QF02_P	YN01_P, YS01_P, FN03_P
7.32	103-65-1	n-Propylbenzene	I	91, 120, 65		QF02_P	FN03_P
7.01	6137-06-0	4-Methyl-2-heptanone	II	43, 58, 85			FN03_P
7.50	620-02-0	5-Methyl-2-furfural	III	110, 53, 81			FN02_P
7.57	100-52-7	Benzaldehyde	I	105, 77, 51	LE01, LE02, LS01, LS02	QF02_P	YN01_P/L, YS01_P/L, FN03_P/L
7.87	98-83-9	Alpha-methylstyrene	I	118, 103, 78			YN01_P, YS01_P, FN01_L, FN03_P
8.19	111-90-0	Diethylene glycol monoethyl ether	I	45, 59, 72	LE01, LE02, LE03, LS01, LS02		
8.35	93-53-8	2-Phenylpropanal	I	105, 134, 91		QF02_P	
8.38	13429-07-7	1-(2-Methoxypropoxy)-2-propanol	III	59, 73, 104	LE02, LS02		
8.58	110-98-5	1,1′-Oxydi-2-propanol	III	45, 89, 59	LE01, LE02, LE03, LS01, LS02		
8.67	104-76-7	2-Ethyl-1-hexanol	I	57, 43, 83			FN03_P
8.98	122-78-1	Phenylacetaldehyde	I	91, 120, 65		QF02_P	FN03_P
9.23	110-03-2	2,5-Dimethyl-2,5-hexanediol	III	43, 59, 70			FN01_P
9.36	96-09-3	Styrene-7,8-oxide	III	91, 119, 63		QF02_P	
9.40	98-86-2	Acetophenone	I	105, 77, 120	LE02, LS02	QF02_P	YN01_P, YS01_P, FN03_P
9.79	821-55-6	2-Nonanone	II	58, 43, 71	LE01		
10.75	28564-83-2	2,3-Dihydro-3,5-dihydroxy-6-methyl-4H-pyran-4-one	III	144, 101, 43			FN02_P
11.18	65-85-0	Benzoic acid	I	105, 122, 77			YN01_P/L, YS01_P/L, FN02_P
11.55	106-32-1	Ethyl caprylate	I	88, 101, 127	LE01, LS01		
12.05	103-11-7	2-Ethylhexyl acrylate	I	55, 70, 83	LE01, LS01		
12.06	122-99-6	Phenoxyethanol	II	94, 138, 77			YN01_P, YS01_P
12.12	67-47-0	5-Hidroxymethyl-2-furfuraldehyde	III	95, 126, 69			FN01_P, FN02_P
12.60	105-60-2	Caprolactam *	III	113, 55, 85	LE01, LE02, LS01, LS02	QF01, QF02_L	YN01_P/L, YS01_L/P, FN01_P/L, FN02_P, FN03_P/L
13.00	104-55-2	Cinnamaldehyde	I	131, 103, 77	LE01, LE02, LS01, LS02		FN03_P/L
13.39	93-56-1	Styrene glycol	I	107, 79, 51			FN03_P
13.49	85-44-9	Phthalic anhydride	III	104, 76, 148			FN01_L, FN03_P
13.81	102-76-1	Triacetin *	I	43, 103, 145		QL01_E	YN01_L, YS01_L
14.00	91-08-7	2,6-Toluene diisocyanate *	III	174, 146, 118		QF02_L	YN01_L, YS01_L, FN02_P, FN03_L
14.10	584-84-9	2,4-Toluene diisocyanate *	III	174, 145, 132		QF02_L	YN01_L, YS01_L
14.09	97-53-0	Eugenol	I	164, 149, 103	LE01, LE02, LE03, LS01, LS02		
15.00	480-63-7	Mesitylene carboxylic acid	I	146, 164, 118			FN02_P/L
15.28	101-81-5	Diphenylmethane	III	167, 153			YN01_L, YS01_L, FN03_L
15.67	719-22-2	2,6-Di-tert-butyl-p-benzoquinone *	II	177, 135, 220	LE03, LS02	QS01_L, QF02_L/P	YN01_P/L, YS01_P/L, FN01_P/L, FN03_P/L
15.78	2607-52-5	2,6-Di-tert-butyl-4-methylene-2,5-cyclohexadienone	II	161, 203, 175		QS01_L, QL01_I	FN02_L
15.81	112-53-8	1-Dodecanol	I	55, 69, 83	LE01, LE02, LS01, LS02		FN03_L
16.22	128-37-0	Butylated hydroxytoluene *	II	205, 220, 177	LE01, LE02, LE03, LS01, LS02	QS01_L/P/I, QL01_I, QF02_L/P	YN01_L, YS01_L, FN01_L, FN02_P/L, FN03_P/L
16.25	96-76-4	2,4-Di-tert-butylphenol	I	191, 206, 57	LE01, LE03, LS01, LS02	QL01_E, QF01	YN01_P/L, YS01_P/L, FN01_P, FN02_L, FN03_L
16.36	93-28-7	Eugenol acetate	I	164, 149, 131			FN03_L
16.43	530-48-3	1,1-Diphenylethylene	III	180, 165, 89		QF01_P	YN01_P, YS01_P, FN03_P
16.60	105-76-0	Dibutyl maleate	I	99, 57, 117		QF02_L	
16.92	5814-85-7	1,2-Diphenylpropane	III	105, 79, 91			YN01_P, YS01_P
17.37	4098-71-9	Isophorone diisocyanate	III	110, 123, 81		QS01_L, QL01_E	
17.37	84-66-2	Diethyl phthalate *	I	149, 177, 150	LE01, LE02, LE03, LS01, LS02	QS01_-/P/I, QF02_L/P	YN01_P/L, YS01_P/L, FN01_P/L, FN02_L, FN03_P/L
17.80	10233-13-3	Isopropyl laurate	I	43, 102, 183			FN02_L
18.02	119-61-9	Benzophenone *	III	105, 77, 182	LE01, LE02, LE03, LS01, LS02	QF02_L/P	
18.27	1081-75-0	1,3-Diphenylpropane *	III	92, 105, 196		QS01_I, QF02_P	YN01_P, YS01_P, FN03_P
18.78	20071-09-4	trans-1,2-Diphenylcyclobutane	III	104, 78, 178		QS01_I, QF02_P	FN03_P
18.98	3910-35-8	1,1,3-Trimethyl-3-phenylindan	III	221, 143, 128	LS01	QF02_L	YN01_P/L, YS01_P/L, FN03_P
19.00	1020-31-1	3,5-Di-tert-butylcatechol	I	207, 222, 179			FN01_P
19.04		Unknown (naphthalene compound structure)		197, 155, 212	LE02, LE03, LS01, LS02	QF02_L/P	
19.51	1620-98-0	3,5-Di-tert-butyl-4-hydroxybenzaldehyde	II	219, 191, 234			FN02_L
19.73	3018-20-0	1-Phenyltetralin	III	130, 180, 208		QS01_I, QF02_P	YN01_P, YS01_P, FN03_P
19.92	1889-67-4	2,3-Dimethyl-2,3-diphenylbutane	III	119, 91, 77	LS01	QF02_L	YN01_P/L, YS01_P/L, FN03_P
20.25	110-27-0	Isopropyl myristate	I	102, 60, 228	LE01, LE02, LS01, LS02		
20.56	1222-05-5	Galoxolide	III	243, 213, 258	LE01, LS01, LS02		
20.70	84-69-5	Diisobutyl phthalate (DIBP) *	I	149, 57, 150	LE01, LE02, LE03, LS01, LS02	QF02_P	YN01_P/L, YS01_P/L, FN01_P/L, FN02_P/L, FN03_P/L
20.78	605-02-7	1-Phenylnaphthalene	III	204, 101, 88		QS01_I, QF02_P	
21.21	82304-66-3	7,9-Di-tert-butyl-1-oxaspiro[4.5]deca-6,9-diene-2,8-dione	III	205, 57, 175	LE01, LE02, LE03, LS01, LS02	QS01_L, QL01_E, QF01, QF02_L/P	YN01_P/L, YS01_P/L, FN01_L, FN02_P/L, FN03_P/L
21.41	112-39-0	Methyl palmitate *	I	74, 87, 43	LE01, LS01, LS02		
21.47	6386-38-5	Benzenepropanoic acid	II	277, 147, 219		QL01_I	FN01_P
21.75	84-74-2	Dibutyl phthalate (DBP) *	I	149, 150, 104	LE01, LE02, LE03, LS01, LS02		YN01_L, YS01_L
22.06	110-36-1	Butyl myristate	I	56, 229, 129			FN01_L
22.47	142-91-6	Isopropyl palmitate	I	43, 102, 256	LE02		
22.98	3524-68-3	Pentaerythritol triacrylate	I	55, 81, 126			FN02_L
23.27	112-62-9	Methyl oleate	I	55, 43, 69		QF02_L/P	FN02_P
23.35	101-68-8	4,4′-Diphenylmethane diisocyanate	III	250, 208, 221		QL01_E	
23.37	103-41-3	Benzyl cinnamate	I	131, 91, 192			FN03_L
23.82	7568-58-3	Tributyl trans-aconitate	I	112, 157, 139		QL01_E	
23.98		Unknown (phthalate compound structure)		149, 150, 86			FN01_L
24.07	629-54-9	Hexadecanamide *	III	59, 72, 43		QS01_P, QL01_E	YN01_L, YS01_L, FN01_P, FN02_L, FN03_L
24.14	111-06-8	Butyl palmitate	I	56, 257, 129			YN01_P/L, YS01_P/L, FN01_L, FN02_L, FN03_P
24.24	959-26-2	Bis(2-hydroxyethyl) terephthalate	I	149, 193, 211		QS01_P	
24.47	65745-83-7	3,6,9,12,15-Oxabicyclo(15,3)heneicosa-1(21),17,19-triene-2,16-dione	III	149, 193, 104			FN01_L
24.59	483-65-8	Retene	III	219, 234, 204	LE01		
24.72	77-90-7	Acetyltributyl citrate (ATBC) *	I	185, 129, 259		QS01_L, QL01_E/I, QF01, QF02_L	YN01_P/L, YS01_P/L, FN01_L, FN02_P/L, FN03_L
24.72	21956-56-9	3,5-Dimethoxystilbene	III	240, 165, 152	LE02, LE03, LS02		
24.90	5776-79-4	1,8-Diazacyclotetradecane-2,9-dione	III	114, 198, 55		QF01	
25.09	13601-88-2	Dehydroabietal	II	159, 173, 129	LE01, LE02, LE03, LS01, LS02		
25.70	1235-74-1	Methyl dehydroabietate	II	239, 141, 129	LE01, LE02, LE03, LS01, LS02		YN01_L, YS01_L, FN02_P
25.76	112-63-0	Methyl linoleate	I	67, 81, 95			FN03_L
26.16	103-23-1	Diethylhexyl adipate (DEHA) *	I	129, 70, 111	LE01, LE03, LS01, LS02		YN01_L, YS01_L, FN03_P/L
26.86-28.39		Unknown (phthalate compound structure)		149, 57, 71	LE01, LS02		
26.68	1740-19-8	Dehydroabietic acid	II	239, 285, 197			YN01_L, YS01_L, FN03_L
27.09	514-10-3	Abietic acid	III	136, 105, 302			YN01_L, YS01_L, FN03_L
27.35	117-81-7	Bis(2-ethylhexyl)phthalate (DEHP) *	I	149, 167, 57	LE03, LS01	QS01_P, QL01_E, QF01, QF02_L	YN01_L, YS01_L, FN01_P/L, FN02_P/L, FN03_L
27.48	56728-02-0	(1-Methyl-2,2-diphenylcyclopropyl)sulfanylbenzene	III	129, 91, 207		QS01_I, QF02_P	
28.38	6197-30-4	Octocrylene *	III	204, 232, 250	LE02, LS01, LS02		FN01_P, FN02_L
28.48	1633-22-3	Di-p-xylylene	III	104, 208, 78		QS01_I, QF02_P	YN01_P, YS01_P, FN03_P
29.11	6422-86-2	Bis(2-ethylhexyl) terephthalate (DEHT) *	I	70, 149, 112	LE02, LS01, LS02	QF01, QF02_L	FN02_L
29.42	112-84-5	Erucamide *	III	59, 72, 43		QS01_L/P, QL01_E/I, QF01	FN01_P, FN02_P/L, FN03_P/L
29.67	111-02-4	Squalene *	I	69, 81, 95	LE01, LE02, LS01, LS02	QS01_P/I, QL01_I, QF01, QF02_L/P	YN01_P/L, YS01_P/L, FN01_P/L, FN02_P/L, FN03_P/L
29.71		Unknown (phthalate compound structure)		149.0232	LE03, LS01		FN01_L
30.58	538-23-8	Glyceryl tricaprylate *	I	127, 57, 201		QS01_P	

* Confirmed with the standard. TR: retention time.

**Table 3:** Method validation parameters results.

Compound	Equation	*r*	LOD (µg/g)	LOQ (µg/g)	Recovery (%) (*n* = 6)			Intermediate Precision (RSD %) (*n* = 6)		
					0.25 µg/g	0.5 µg/g	1 µg/g	0.25 µg/g	0.5 µg/g	1 µg/g
DEP	Y = 1.6592x + 0.1137	0.9940	0.01	0.025	112	114	102	16.1	8.36	2.50
BP	Y = 0.5995x + 0.0401	0.9941	0.05	0.1	107	104	102	14.9	7.67	2.03
1,3-DPP	Y = 1.4391x + 0.0164	0.9932	0.025	0.05	88.8	80	78.4	5.03	1.73	7.01
DIBP	Y = 2.3345x + 0.0726	0.9981	< 0.005	0.005	112	123	124	5.40	2.80	1.61
DBP	Y = 2.323x − 0.0116	0.9984	< 0.005	0.005	120	124	123	8.52	0.98	4.26
DEHP	Y = 0.9686x − 0.0019	0.9900	< 0.005	0.005	123	124	116	1.62	1.04	7.51
DEHT	Y = 0.3052x − 0.009	0.9909	0.025	0.05	96.2	89.6	98.9	16.0	15.9	18.2

**Table 4:** Mean concentrations of migrants in pooled food samples and estimated dietary exposure of the Spanish child and adolescent population.

Compound	Concentration (µg/g)			Dietary Exposure µg/kg bw per Day					
				Mean			P95		
	12–35 Months	3–9 Years	10–17 Years	12–35 Months	3–9 Years	10–17 Years	12–35 Months	3–9 Years	12–35 Months
DEP	0.03 ± 0.005	0.03 ± 0.005	0.03 ± 0.004	1.19	0.82	0.47	6.24	3.63	2.29
BP	˂LOQ	˂LOQ	˂ LOQ	2.25	1.60	0.74	11.80	7.06	3.61
1,3-DPP	˂LOQ	˂LOQ	˂ LOQ	1.13	0.80	0.37	5.88	3.53	1.81
DIBP	0.08 ± 0.01	0.05 ± 0.004	0.10 ± 0.01	3.49	1.61	1.55	18.20	7.10	7.51
DBP	0.10 ± 0.003	0.09 ± 0.002	0.16 ± 0.04	4.40	2.95	2.42	23.00	13.00	11.80
DEHP	0.09 ± 0.03	0.07 ± 0.03	0.09 ± 0.02	4.07	2.13	1.35	21.30	9.45	6.54
DEHT	˂LOQ	˂LOQ	˂LOQ	1.13	0.80	0.37	5.88	3.53	1.81

LOQ: BP = 0.1 µg/g; 1,3-DPP = 0.05 µg/g; DEHT = 0.05 µg/g. For analytical results < LOQ (non-quantifiable), the LOQ/2 was considered to estimate the dietary exposure.
